# GENDER DIFFERENCES IN YEARS OF GOOD LIFE AMONG OLDER ADULTS IN INDIAN SUBNATIONAL LEVEL POPULATION

**DOI:** 10.1093/geroni/igae098.3511

**Published:** 2024-12-31

**Authors:** Ruchira Chakraborty

**Affiliations:** International Institute for Population Sciences, Mumbai, Maharashtra, India

## Abstract

The study investigates the heterogeneities in healthy life among older-adults in India, using a novel concept of counting ‘expected years of good life’. Grounded in Amartya Sen’s ‘capable longevity’ approach, the index of YoGL (Years of Good Life) is an amalgamation of objective and subjective dimensions of health. A year is counted as ‘good’ if a person is simultaneously out of poverty, free from severe activity limitations and cognitive impairments, and experiences positive life satisfaction. After attaining a certain age, remaining good life years within life expectancy are computed by employing Sullivan’s method using Longitudinal Ageing study in India,2017-18 (LASI wave-I) dataset. The results show, at age 50, YoGL for male is 13.9 years (55.6% of remaining life expectancy) and for females, 11.3 years (41.7% of remaining life expectancy). Supporting the ‘gender-health-mortality paradox’, results reveal that women’s advantage in life expectancy doesn’t translate into equal advantage in ‘good-years’ for all older age-groups. Inter-state variability is higher in terms of YoGL, than in life expectancy; from 64% years counts as ‘good life’ in Punjab to 34% of remaining years as ‘good life’ in Odisha. Regression decomposition indicates functional limitations contributes 32%, cognitive impairment 20% and birth-cohorts contributes around 18% in explaining the regional heterogeneity.

